# House Sparrows Do Not Constitute a Significant *Salmonella* Typhimurium Reservoir across Urban Gradients in Flanders, Belgium

**DOI:** 10.1371/journal.pone.0155366

**Published:** 2016-05-11

**Authors:** Lieze Oscar Rouffaer, Luc Lens, Roel Haesendonck, Aimeric Teyssier, Noraine Salleh Hudin, Diederik Strubbe, Freddy Haesebrouck, Frank Pasmans, An Martel

**Affiliations:** 1 Department of Pathology, Bacteriology and Avian Diseases, Faculty of Veterinary Medicine, Ghent University, Merelbeke, Belgium; 2 Department of Biology, Terrestrial Ecology Unit, Ghent University, Ghent, Belgium; 3 Department of Biological Sciences, Faculty of Science & Mathematics, Universiti Pendidikan Sultan Idris, Perak, Malaysia; Justus-Liebeig University Giessen, GERMANY

## Abstract

In recent decades major declines in urban house sparrow (*Passer domesticus*) populations have been observed in north-western European cities, whereas suburban and rural house sparrow populations have remained relatively stable or are recovering from previous declines. Differential exposure to avian pathogens known to cause epidemics in house sparrows may in part explain this spatial pattern of declines. Here we investigate the potential effect of urbanization on the development of a bacterial pathogen reservoir in free-ranging house sparrows. This was achieved by comparing the prevalence of *Salmonella enterica* subspecies *enterica* serotype Typhimurium in 364 apparently healthy house sparrows captured in urban, suburban and rural regions across Flanders, Belgium between September 2013 and March 2014. In addition 12 dead birds, received from bird rescue centers, were necropsied. The apparent absence of *Salmonella* Typhimurium in fecal samples of healthy birds, and the identification of only one house sparrow seropositive for *Salmonella* spp., suggests that during the winter of 2013–2014 these birds did not represent any considerable *Salmonella* Typhimurium reservoir in Belgium and thus may be considered naïve hosts, susceptible to clinical infection. This susceptibility is demonstrated by the isolation of two different *Salmonella* Typhimurium strains from two of the deceased house sparrows: one DT99, typically associated with disease in pigeons, and one DT195, previously associated with a passerine decline. The apparent absence (prevalence: <1.3%) of a reservoir in healthy house sparrows and the association of infection with clinical disease suggests that the impact of *Salmonella* Typhimurium on house sparrows is largely driven by the risk of exogenous exposure to pathogenic *Salmonella* Typhimurium strains. However, no inference could be made on a causal relationship between *Salmonella* infection and the observed house sparrow population declines.

## Introduction

*Salmonella enterica* subspecies *enterica* serotype Typhimurium has the potential to cause disease outbreaks in Passeriformes. In Britain, definite phage types (DT)40, DT56(v) and DT160, accounted for the majority of passerine salmonellosis incidents, most often recognized in greenfinches (*Chloris chloris*) and house sparrows (*Passer domesticus*) [1–3]. Outbreaks of salmonellosis in Passeriformes occur mostly during the winter period [1;2;4;5] with sometimes marked annual variation in salmonellosis incidents between winter periods of consecutive years [2;3;5]. Harsh weather conditions [6] and contaminated foraging areas [7–9], sometimes related to supplemental feeding [10;11], have been associated with a higher prevalence of *Salmonella* spp. [6–11]. Although some phage types of *Salmonella* Typhimurium are considered host adapted, DT2 and DT99 in pigeons (*Columba livia*) [12], DT40 and DT56(v) in passerines [13], the latter two phage types have been isolated from captive birds and mammals and have been linked to disease in humans [1;3;14–16]. In this perspective, most of the studies on prevalence and epidemiology of *Salmonella* spp. in free-living birds have been performed in the surroundings of farms in order to evaluate food safety and human health risks [7;9;17], or have been related to disease in animals or humans [3;14;15]. Other studies, none of which were conducted in Belgium, assessed the presence of pathogenic bacteria in moribund birds, or dead birds submitted for necropsy, whether or not related to epidemics in wild birds [1;2;5;11;15]. While these studies provide important insights in the epidemiology and pathogenesis of these bacteria, they cannot be used to estimate the prevalence of long-term carrier birds. Few studies have been performed to assess the prevalence of *Salmonella* spp. in apparently healthy migrating and non-migrating wild Passeriformes, not specifically related to ongoing disease outbreaks. A low prevalence (≤ 2%) of *Salmonella* spp. was demonstrated in these studies [8;9;11;18–20]. Since host-adapted *Salmonella enterica* strains could potentially reduce the reproduction success in their respective reservoir hosts [21;22], it is important to understand to what extent passerines are indeed long term carriers of *Salmonella* Typhimurium, as birds in general have already been appointed as potential reservoirs for *Salmonella enterica* subspecies *enterica* [8;23;24].

Little research has been performed to specifically assess the differences in prevalence of *Salmonella enterica* subspecies *enterica* in wild passerines inhabiting urban versus rural environments [20]. Previous studies have suggested that the prevalence of *Salmonella enterica* subspecies *enterica* may depend on microclimate differences between urban (heat island effect) and rural areas [20;24]. As such, this pathogen might be partly responsible for discrepant population dynamics in avian hosts from urban and rural areas, such as observed in house sparrows (*Passer domesticus*). In recent decades, urban populations of this species have indeed suffered dramatic declines throughout north-western Europe and south-east Asia, whereas suburban and rural populations have remained relatively stable or are recovering from previous declines [25;26]. Understanding the role, if any, of house sparrows as *Salmonella* Typhimurium reservoirs is important for understanding infection and disease dynamics. This might help to explain the massive population declines observed, possibly related to disease outbreaks during the winter and lower reproduction successes in spring.

We here assess the prevalence of *Salmonella* Typhimurium in apparently healthy house sparrows along urban gradients, in order to reveal potential correlates with the ongoing population declines in urban areas. To achieve this goal, feces and blood samples of house sparrows, collected in urban, suburban and rural populations, were tested for the presence of *Salmonella* Typhimurium and anti-*Salmonella* antibodies respectively. In addition, a total of twelve deceased house sparrows, obtained from the bird rescue centers of Ostend and Merelbeke, and submitted for necropsy, were tested for the presence of *Salmonella* Typhimurium.

## Materials and Methods

Since *Salmonella* Typhimurium outbreaks in passerines are reported mostly during the winter period [1;2;4;5;11;15;24], feces and blood samples of 364 individual house sparrows were collected between September 11^th^ and December 20^th^, 2013 (first sampling) and between January 10^th^ and March 28^th^ 2014 (second sampling). Samples were collected in 36 house sparrow populations located in 9 urban, 9 suburban and 18 rural regions clustered pairwise around the Flemish cities of Ghent, Antwerp and Louvain, every population being sampled at least once per sampling period. House sparrows are treated as species of Least Concern on the IUCN Red List of Threatened Species (http://www.iucnredlist.org/), and all ringers involved in this study were holders of a scientific ringing certificate issued annually by the Agency for Nature and Forest. All sparrows were captured on private land for which oral permission was granted by the respective land owners. All trapping and sampling protocols were approved by the Ethical Committee VIB Ghent site (EC2013-027).

The level of built up area (BU) in circular plots around each trapping site was calculated from GIS layers at two nested scales, i.e. a local scale (radius of 400m) and a landscape scale (radius of 1600m) (Large-scale Reference Database (LRD)) the former corresponding to the average home-range size of Flemish house sparrows [25;27]. Built up values for the three urbanization classes were empirically set as “urban” >13% BU; “suburban” 5–13% BU; “rural” <5% BU, and neighboring populations were at least 1km apart. The landscape scale was used for the classification of the urbanization levels, whereas the local scale provides more detail regarding the urbanization of the center of each individual class, being the direct habitat of the house sparrows (S1 Table).

House sparrows were captured with standard mist nets after which each bird was individually put in an autoclaved cotton bag (approved by the Ethical Committee VIB Ghent site: EC2013-027). Feces were collected in sterile micro centrifuge tubes, 50 μl blood was collected in 200μl absolute ethanol and each individual was sexed, measured and equipped with a unique metal ring before being released at its original trapping site. The Scaled Mass Index (SMI) of the house sparrows was calculated [28], in order to have an estimation of the body condition of the birds, and compared to the different urbanization levels at both scales (400m and 1600m radius) using ANOVA in R.

The ISO 6579:2002 method [29], for the isolation of different *Salmonella* serotypes including *Salmonella* Typhimurium, was initiated within 24 hours of sampling. In summary, the fecal samples were pre-enriched overnight at 37°C in non-selective Buffered peptone water (Oxoid, Hampshire, UK), after which the samples were simultaneously added to selective “Tetrathionate brilliant-green enrichment broth for Microbiology’ (Merck, Belgium) and “Rappaport Vassiliadis medium with Soya Peptone Broth” (Oxoid, Hampshire, UK) for overnight enrichment at 37°C and 41°C respectively. Xylose Lysine Deoxycholate agar (Oxoid, Hampshire, UK) and Brilliant Green agar (BGA) (Oxoid, Hampshire, UK), incubated overnight at 37°C, were used for plating out the samples after the enrichment procedures.

Indirect ELISA was performed on the blood samples. The preparation of ELISA plates was conducted according to [30] using a formol-inactivated *Salmonella* Typhimurium DAB69 (pigeon strain) for plate-coating. Before initiation of the indirect-ELISA the plates were washed with a 1% skim milk powder solution in distilled water. The blood samples, stored in ethanol were thoroughly vortexed, after which 100μl of a 1/100 dilution of the samples in Sample Diluent Buffer (0.6 g NaH2PO4·2H2O, 5.6 g NaH2PO4. 12H2O, 0.5 ml Tween 20 (Merck, Germany), 12.5 g NaCl, 22g skim milk powder, 1000ml distilled water) was added to the wells. The plate was incubated for 1 hour at 37°C after which the plate was washed three times with washing buffer (0.6 g NaH2PO4·2H2O, 5.6 g NaH2PO4. 12H2O, 0.5 ml Tween 20, 12.5 g NaCl, 1000ml distilled water). A 1/1000 dilution of Polyclonal Goat Anti-Bird IgG (H+L)-horseradish peroxidase (HRP) conjugate (Cat-number: 90520, Alpha Diagnostics Intl. Inc., San Antonio, Texas, USA), reactive against sparrow and dove antibodies, was added to the wells. The plates were incubated at 37°C for 1 hour and washed three times, after which 100μl of 3,3′,5,5′-Tetramethylbenzidine (TMB) Liquid Substrate System for ELISA (Sigma Aldrich Chemie Gmbh, Steinheim Germany) was added. After 15min incubation at room temperature in a dark environment, the reaction was stopped using stop reagent for TMB substrate (Sigma Aldrich Chemie Gmbh, Steinheim Germany), and the optical density was measured (450nm). Blood in ethanol of *Salmonella* infected pigeons (infected with the DAB69 strain) served as a positive control. The control blood was obtained from another study approved by the Ethical Committee of the Faculty of Veterinary Medicine and Bioscience Engineering, Ghent University (EC2014/96). The cut-off point for the optical density (OD) was calculated as the mean OD from three *Salmonella-*free pigeons, calculated from an entire 96-well plate, plus three times the standard deviation (0.238). All measurements were performed in duplicate. The pigeons used for calculation of the cut-off value for the OD were ascertained *Salmonella*-free, since they were retrieved at 4 weeks of age from a *Salmonella-*negative colony, where-after they were housed strictly separately from other pigeons following the bio-security measurements and were tested for several weeks by the use of ISO 6579:2002 method on mixed feces. In addition the individual pigeons were screened for the presence of *Salmonella* Typhimurium by performing bacteriology on cloacal swabs and a rapid slide agglutination test on serum. The positive control consisted of an experimentally *Salmonella* Typhimurium infected pigeon.

During 2013–2014, bird rescue centers were asked to transfer deceased house sparrows to the lab facilities of the Faculty of Veterinary Medicine (Ghent University), for necropsy. A total of twelve birds, received between May 2013 and December 2014 were tested for the presence of *Salmonella* Typhimurium. The entire intestinal tract, heart, lungs, liver, spleen, kidneys, reproductive organs and brains were checked for abnormalities. If obvious lesions were present, these were aseptically swabbed, prior to enrichment, and immediately plated out onto BGA and Columbia agar with sheep blood plus (COLS) (Oxoid, Wesel, Germany) for overnight incubation at 37°C and onto MacConkey agar (Oxoid, Hampshire, UK) for overnight incubation at 30°C. Direct microscopic investigation was conducted on the intestinal content. Unless postmortem decay was too advanced, cytology was performed on the liver, spleen, kidney and lungs and a separate enrichment according to the ISO 6579:2002 protocol was initiated for the intestinal content, liver, spleen, and organs with lesions.

If fecal or autopsy samples were positive for *Salmonella* spp., these *Salmonella* spp. were further analysed by serotyping at the ‘Belgian Scientific Institute of Public Health (WIV-ISP)’ and by phage typing at the ‘Bacteriology Reference Department of the Public Health of England (BRD-PHE)’.

In order to estimate the probability of absence of *Salmonella* serotypes in our population, we applied the epi.detectsize function of the R library ‘epiR’ [31]. This test determines the number of individuals that need to be randomly sampled to declare a population free from a pathogen at a certain confidence level. The test is based on the pathogen prevalence level we want to be able to reveal, the population size and test sensitivity and specificity. Based on literature regarding *Salmonella* prevalence among passerines, we can expect the between- and within-population prevalence to be lower than 2% [8;9;11;18–20]. Average sparrow population size in our study area was estimated at about 25 birds. The analyses are based on the highly sensitive ISO 6579:2002 method outlined above. This test is characterised by a sensitivity of at least 0.90 and a specificity of at least 0.99 [32;33]. ISO based analyses yield a conservative estimate of the power and precision of our analyses. In addition, we used the truePrev function of the R library ‘prevalence’ [34] to obtain a Bayesian estimate of true prevalence from apparent prevalence obtained by testing individual samples, using the sensitivity and specificity values mentioned above.

## Results

In total, feces and blood of 364 house sparrows were screened for the presence of *Salmonella* Typhimurium and the presence of anti-*Salmonella* antibodies. The house sparrows consisted of 42.6% female birds, 57.1% male birds and 1 undefined young house sparrow, which belonged to urban house sparrow populations (28.3%), suburban populations (21.15%), and to rural populations (50.55%). Nineteen birds were recaptured within or between both sampling periods, from which 19 fecal and 11 blood samples were obtained, respectively. The recaptured birds all originated from the same house sparrow population as the one they were first captured from. Sparrow SMI (95%CI: mean = 27.68g+/-3.86g) did not vary across urbanization gradients (1600m scale: ANOVA F_1,352_ = 2.19, P-value = 0.14; 400m scale: F_1,352_ = 1.70, P-value: 0.19) and all trapped individuals appeared healthy, with the exception of one bird with multiple severe skin lesions which was moribund and euthanized for welfare reasons. This bird was diagnosed with pox-virus based on the macroscopic cutaneous lesions and the detection of typical intracytoplasmic Bollinger bodies within the epidermal cells. *Salmonella* Typhimurium was not isolated from any of these fecal samples. One house sparrow (0.27%) trapped in the city of Ghent (Ghent: 51,052083 /3,694134: U), proved to be positive for anti-*Salmonella* antibodies (mean OD: 0.388).

Statistical analyses show that to be 95% certain that *Salmonella* Typhimurium is not present in the study area (i.e. prevalence < 1%), if all tests were to be negative, we would need to sample 12 sparrows from 18 populations (216 sparrows in total), which is close to our actual sampling (364 birds from 36 populations). Since this study utilizes a stratified random sampling methodology along urbanization gradients across Flanders, our results can be regarded as representative for the whole region. House sparrows are unlikely to number more than one million birds in Flanders [35], and calculations show that a minimum of 331 sparrows need to be sampled to confirm the absence of *Salmonella* serotypes in Flanders. Bayesian analyses showed we can be 95% certain that the true prevalence in Flanders varies between 0 and 1.3%.

Twelve deceased house sparrows, received from the bird rescue centers of Merelbeke (7) and Ostend (5), were necropsied and screened for the presence of *Salmonella* Typhimurium. Two of these individuals, collected in the city of Ostend, showed macroscopically visible granulomas (1.5mm and 3mm diameter) in the cerebrum, histologically consisting of an accumulation of heterophilic granulocytes and macrophages. Ziehl Nielsen-, PAS- and Gram-staining of the granulomas yielded negative results for *Mycobacterium* spp., fungi and Gram-positive bacteria respectively, Gram-negative bacilli were not observed. Both house sparrows however tested positive for *Salmonella* Typhimurium, which was isolated in pure culture from the granulomas in the brains. Serotyping and phage typing revealed the presence of *Salmonella* Typhimurium var. Copenhagen (O:1,4,12) DT195 and a pigeon specific phage type DT99. The former bird also showed a black intestinal content suggestive for hemorrhagic diathesis, while the latter house sparrow was found to be positive for cestodes using direct microscopic investigation of intestinal content. Because of the postmortem decay, the other organs, besides the brains, were not subjected to histology. Nevertheless cytology of the liver, spleen and lungs was performed and did not reveal any *Atoxoplasma*-inclusions, whereas a slight infiltration of granulocytes and macrophages was present in the lungs of the house sparrow infected with DT99. No other major abnormalities were detected. Six house sparrows brought in for necropsy, died due to trauma (2 cases), coccidiosis (2 cases), predation (1 case) or predation with additional *Pasteurella multocida* infection (1 case) while four other individuals died due to unknown reasons. Unfortunately, no information regarding the habitat-type nor the level of urbanization was available for the necropsied house sparrows.

## Discussion

Since the onset of the severe population declines in rural and urban house sparrows, researchers have been searching for possible explanations [reviewed in 25]. Loss of nesting and foraging areas, changes in socio-economic status, electromagnetic radiation, predation, depletion of food resources, pesticides, herbicides, the use of unleaded petrol and pathogens have all been suggested to cause these declines, either separately or in synergy [25;26;36–38]. While the impact that pathogens have on the population health when present in sub-lethal doses or in carrier birds is not very well known, it could potentially depend on infection pressure, which has been suggested to be higher in urban environments [24;39].

Our findings suggest that house sparrow populations across Flemish urban gradients, during the winter of 2013–2014, do not constitute a considerable *Salmonella* Typhimurium reservoir, such that birds could overall be considered naïve to infection. Not isolating *Salmonella* Typhimurium from the feces and a seroprevalence for *Salmonella* spp. of 0.27% in the house sparrows screened during this study, indeed suggests a very low prevalence of *Salmonella* Typhimurium. Bayesian estimates confirm that true *Salmonella* Typhimurium prevalence is unlikely to be higher than 1.3%. Annual variation in salmonellosis incidents [2;3] should however be kept in mind when interpreting the results, since the study was limited to a single winter period (2013–2014). Based on our results, no patterns regarding *Salmonella*-prevalence in house sparrow populations along an urban-rural gradient could be demonstrated and no inference could be made on a causal relationship between *Salmonella* and the house sparrow declines. Despite the lack of historical data regarding the prevalence of *Salmonella* in apparently healthy Passeridae in Belgium, our findings are consistent with those obtained from house sparrow populations in Northern Spain [9], which investigated the difference in prevalence of *Salmonella* in house sparrows living close to or far from pig premises, and in Ohio [8], which focused on house sparrows and other birds in the surrounding of human settlements. Both studies detected low prevalence of *Salmonella* spp. in these birds [8;9], especially in birds inhabiting areas far from pig premises [9]. Occasional detection of *Salmonella* in feces from apparently healthy house sparrows [8;9;11], which were not corroborated by follow up data, may reflect mechanical or temporal carriage after foraging in contaminated areas, or could indicate the presence of *Salmonella-*excreting birds still in the incubation period of the disease, rather than the demonstration of the presence of actual *Salmonella* carrier birds.

Anti-*Salmonella*-antibodies were detected in one of 364 house sparrows. To the authors’ knowledge, this is the first study to detect antibodies against *Salmonella* spp. in apparently healthy wild passerines. Serum-IgG-antibodies have proved to provide a good indication of *Salmonella* Typhimurium-infection since they increase within 2 weeks of primary infection and as they can persist in the blood for several months [40–42]. Despite this knowledge, the onset of the antibody response and the height of the antibody titer depends on the maturity of the immune system, on whether or not the infection is a primary infection or a reinfection and on the susceptibility of the bird to *Salmonella* Typhimurium [40–42]. The main advantage of ELISA, when performed in conjunction with isolation methods and recapture of birds, is that ELISA could provide a better assessment of the prevalence of long term carriers/survivors and could aid in the detection of intermittent shedders, however, more research is needed for the accurate interpretation of the results.

Two out of 12 deceased house sparrows sent for autopsy tested positive for *Salmonella* Typhimurium var. Copenhagen (O:1,4,12) DT99 and DT195, isolated from granulomatous brain lesions. Although the sole demonstration of these cerebral lesions, without concurrent hepatomegaly, splenomegaly and granulomatous lesions in the upper alimentary tract, is not typical for a *Salmonella* Typhimurium infection in passerines, histological evidence of encephalitis has previously been demonstrated in passerines and brain abscesses have been recognized in pigeons in the past [15;21;43]. Phage type DT195 has been shown to be pathogenic for a variety of animals including humans [44;45]. DT99, on the contrary, is regarded a pigeon-adapted variant of *Salmonella* Typhimurium [12] which has previously caused morbidity and mortality in mice [23] as well as in passerines [5]. Since *Salmonella* Typhimurium DT99 circulates endemically in feral pigeons that can reach high local densities in urbanized areas [23], feral pigeons constitute a potential source for *Salmonella* Typhimurium DT99 associated disease in passerines in urbanized areas.

## Conclusion

These results suggest the apparent absence (prevalence: <1.3%) of a *Salmonella* Typhimurium reservoir in apparently healthy house sparrows and an association of *Salmonella* Typhimurium infection with clinical disease which is most likely driven by the risk of exogenous exposure to pathogenic *Salmonella* Typhimurium strains. However, no inference could be made on a causal relationship between *Salmonella* and the house sparrow population declines.

## Supporting Information

S1 Table Urbanization level around the sampled house sparrow populations. (DOCX)
