# Supplementary material for: House Sparrows Do Not Constitute a Significant Salmonella Typhimurium Reservoir across Urban Gradients in Flanders, Belgium
Source: PLoS One. 2016 May 11;11(5):e0155366. doi: 10.1371/journal.pone.0155366 (PMC4864353; doi:10.1371/journal.pone.0155366)
Supplement: S1 Table — (DOCX) [file pone.0155366.s001.docx]

| Region | House sparrow Populations | Longitude | Latitude | BU (400m) | BU (1600m) | R: Rural  S: Suburban  U: Urban |
| --- | --- | --- | --- | --- | --- | --- |
| Ghent | Ghent 1 | 3,720391 | 51,056024 | 52,5089082873827 | 38,9889169006724 | U |
| Ghent | Ghent 2 | 3,694134 | 51,052083 | 10,5546454283718 | 18,2861465334554 | U |
| Ghent | Oudenaarde 1 | 3,609749 | 50,860936 | 17,2268912639568 | 17,1650181876377 | U |
| Ghent | Oudenaarde 2 | 3,593535 | 50,868613 | 2,47150351196089 | 9,67964616031078 | S |
| Ghent | Beervelde 1 | 3,840934 | 51,07531199 | 15,1004086994531 | 7,33275445345039 | S |
| Ghent | Beervelde 2 | 3,865265 | 51,06631399 | 5,70731560658718 | 4,64037566494428 | R |
| Ghent | Hillegem 1 | 3,860109 | 50,8971 | 12,6206375669135 | 4,68761974639781 | R |
| Ghent | Hillegem 2 | 3,853673 | 50,877882 | 2,34957526967579 | 4,65574083751064 | R |
| Ghent | Kalken 1 | 3,922065 | 51,0363899 | 11,3788471621377 | 4,68000690368939 | R |
| Ghent | Kalken 2 | 3,931397 | 51,017538 | 1,82528392103871 | 3,76496870958661 | R |
| Ghent | Melsen 1 | 3,703454 | 50,9568099 | 9,78172618904255 | 3,85064062844068 | R |
| Ghent | Melsen 2 | 3,718976 | 50,933968 | 3,22161949106561 | 2,14917599536363 | R |
| Louvain | Louvain 1 | 4,70604 | 50,872827 | 86,88396441 | 56,60641218 | U |
| Louvain | Louvain 2 | 4,689437 | 50,890387 | 10,04554235 | 16,95865168 | U |
| Louvain | Tienen 1 | 4,940661 | 50,808948 | 37,73076043 | 17,88324983 | U |
| Louvain | Tienen 2 | 4,929433 | 50,818505 | 7,805795784 | 10,84407227 | S |
| Louvain | Overijse 1 | 4,535616 | 50,772298 | 20,48178196 | 8,355626995 | S |
| Louvain | Overijse 2 | 4,524511 | 50,783 | 4,628883327 | 8,96950102 | S |
| Louvain | Wezemaal 1 | 4,753309 | 50,94787 | 14,87252171 | 5,406645931 | S |
| Louvain | Wezemaal 2 | 4,729571 | 50,962186 | 3,38811682 | 4,688171966 | R |
| Louvain | Houwaart 1 | 4,86241 | 50,934496 | 5,781088869 | 1,721616962 | R |
| Louvain | Houwaart 2 | 4,83591 | 50,92298 | 0,963359571 | 1,53357915 | R |
| Louvain | Kerkom 1 | 4,868131 | 50,858065 | 6,335537293 | 2,033314027 | R |
| Louvain | Kerkom 2 | 4,885316 | 50,839371 | 2,068461673 | 1,837974521 | R |
| Antwerp | Antwerp 1 | 4,415069 | 51,193798 | 30,42215903 | 24,63842561 | U |
| Antwerp | Antwerp 2 | 4,375288 | 51,21827 | 16,28994223 | 7,575152362 | S |
| Antwerp | Mechelen 1 | 4,468846 | 51,016254 | 29,61879254 | 22,15895592 | U |
| Antwerp | Mechelen 2 | 4,498381 | 51,020235 | 11,41874916 | 16,54371574 | U |
| Antwerp | Lint 1 | 4,496199 | 51,126115 | 20,84978166 | 11,86710032 | S |
| Antwerp | Lint 2 | 4,5158 | 51,135978 | 4,095046473 | 4,346261442 | R |
| Antwerp | Ruisbroek 1 | 4,329907 | 51,086121 | 12,62777236 | 6,143232192 | S |
| Antwerp | Ruisbroek 2 | 4,299856 | 51,089197 | 0,97506915 | 3,098021933 | R |
| Antwerp | Herenthout 1 | 4,732248 | 51,132441 | 6,244653976 | 2,817679332 | R |
| Antwerp | Herenthout 2 | 4,71637 | 51,11956 | 0,757206481 | 1,905847648 | R |
| Antwerp | Pulderbos 1 | 4,69979 | 51,222888 | 9,004842326 | 3,697960746 | R |
| Antwerp | Pulderbos 2 | 4,708955 | 51,232311 | 1,260490826 | 2,569700122 | R |

**S1 Table: Urbanization level around the sampled house sparrow populations:**

Overview of the level of Built Up Area (BU) at two nested scales (400m and 1600m radius) around the pairwise clustered house sparrow populations in the region of Ghent, Louvain and Antwerp. For each house sparrow population the exact coordinates are given (Longitude and Latitude). The classification of the urbanization levels in “Urban”, “Suburban” and “Rural” is based on the BU at 1600m radius.
